# Infectious alphavirus production from a simple plasmid transfection+

**DOI:** 10.1186/1743-422X-8-356

**Published:** 2011-07-19

**Authors:** J Jordan Steel, Brittney R Henderson, Siddhi BC Lama, Ken E Olson, Brian J Geiss

**Affiliations:** 1 Department of Microbiology, Immunology, and Pathology, 1682 Campus Delivery, Colorado State University, Fort Collins, CO 80523 USA; 2 Department of Biochemistry and Molecular Biology, 1870 Campus Delivery, Colorado State University, Fort Collins, CO 80523 USA

**Keywords:** Sindbis, Alphavirus, Virus production, Gateway

## Abstract

We have developed a new method for producing infectious double subgenomic alphaviruses from plasmids transfected into mammalian cells. A double subgenomic Sindbis virus (TE3'2J) was transcribed from a cytomegalovirus PolII promoter, which results in the production of infectious virus. Transfection of as little as 125 ng of plasmid is able to produce 1 × 10^8 ^plaque forming units/ml (PFU/ml) of infectious virus 48 hours post-transfection. This system represents a more efficient method for producing recombinant Sindbis viruses.

## Background

Alphaviruses (Family: *Togaviridae*) are used extensively in molecular biology as tools for gene expression and delivery [1]. Alphaviruses can infect a wide range of species and have small manipulable genomes that can encode and express heterologous genes [2,3]. Alphaviruses possess a positive sense capped RNA that is approximately 11.6 kb in length. The 5' end of the viral RNA is translated into 4 nonstructural proteins (nsP 1-4) which are involved in replicating the viral genome. A negative strand RNA is replicated from the full-length positive strand viral RNA that contains a subgenomic promoter (SGP) that drives transcription of the 26S subgenomic RNA. The subgenomic RNA encodes the viral structural proteins (Capsid, E3, E2, 6K, and E1) necessary for virion assembly [4]. The SGP has previously been duplicated in the viral genome, allowing for heterologous genes to be expressed from the virus is the same fashion as the viral structural proteins [5]. Heterologous genes that have been engineered into alphavirus genomes include fluorescent proteins, luciferases, cellular proteins, antisense RNAs, and ribozymes [6-12]. Engineering a heterologous gene or RNA behind the second subgenomic promoter allows for the production of a fully infectious virus simultaneous with the expression of the heterologous gene in a wide range of species.

The current method used to create a recombinant double-subgenomic virus that expresses a heterologous gene is somewhat inefficient. To insert the gene of interest (GOI) into the virus, the viral infectious clone plasmid is digested with a unique restriction enzyme and the PCR amplified GOI is restriction enzyme digested and ligated into the virus infectious clone plasmid. This approach usually results in the GOI ligating in either the sense or antisense orientation, requiring screening of the resulting clones for the orientation of the insert. Of additional concern with single-site restriction cloning is multiple copies of the GOI ligating into the virus infectious clone plasmid if small inserts are used. Once a clone with the GOI in the correct orientation has been identified and sequenced, the plasmid is linearized using a unique restriction site at the end of the viral genome to allow for run-off RNA transcription. Several micrograms of phenol-chloroform extracted plasmid DNA is used in *in vitro *RNA transcription reactions with a nucleotide cap analog to generate capped viral RNAs. The RNA is then either electroporated into cells or transfected with chemical or liposomal RNA transfection reagents, and virus is collected from culture media 24-72 hours later.

Several points in this process reduce efficiency and increase time of virus production. Insertion of a GOI into the viral genome by restriction cloning is relatively inefficient due to the need to screen for insert orientation. *In vitro *RNA transcription kits that are commonly used are expensive and generally result in low yields of full length capped RNAs (B. Geiss, personal observation). Additionally, phage DNA-dependent RNA polymerases (such as T7 and SP6) have low fidelity and can result in quasi-species from the *in vitro *transcription reaction [13]. Electroporation of cells with RNA requires large numbers of cells (1-5 × 10^6 ^cells/electroporation), is sensitive to salt concentration that can damage cells during electroporation, and require specialized equipment not always available in laboratories. Chemical and liposomal RNA transfection has been used more recently to avoid using electroporation, but RNA degradation during transfection is still a concern.

To make alphavirus expression systems easier to use and more accessible to researchers, we have developed virus expression plasmids that are simple to manipulate and can rapidly and inexpensively produce infectious virus. Building on our previous work with Sindbis virus replicon expression plasmids [14], we generated a double-subgenomic Sindbis virus expression plasmid that transcribes RNA from a cytomegalovirus (CMV) PolII promoter and cleaves the RNA at the 3' end of the viral genome similar to plasmid-based replicon expression systems [14-16]. In addition, we have developed variants of this system that utilize recombination technology to rapidly and efficiently insert a GOI into the virus in the desired orientation. The negative and positive selection capability of the Gateway cloning system makes it attractive for rapid GOI cloning. Using this system we have produced several reporter gene expressing viruses and demonstrate their use in cell culture.

## Methods

### Plasmid Construction

The base TE/3'2J Sindbis virus expression plasmid (pBG167) was constructed by digesting a TE/3'2J replicon expression plasmid pBG68 [14] with HpaI and XbaI restriction enzymes and ligating the vector with T4 DNA ligase to a 4631 bp XbaI/HpaI fragment from the pTE/3'2J infectious clone [17]. pBG218 was created by ligating NheI flanked GFP open reading frame into the unique XbaI site in pBG167. The orientation of the GFP insert was verified by sequencing with BG626 (5' CACCTCTAGACCATGGATCC) and BG583 (5' CTAGATAAATGGTTAATATAGT). pBG167-based recombination ready plasmids were generated by ligating a PCR amplified attR1/attR2 recombination cassette from Gateway pDEST32 (Invitrogen) into pBG167. BG121 (5' CATGGCTAGCACAAGTTTGTACAAAAAAGCTGAACG) and BG122 (5' CATGGCTAGCACCACTTTGTACAAGAAAGCTGAACG) contain NheI restriction sites, and were used to ligate the recombination cassette into XbaI digested pBG167 and were transformed into ccdB resistant DB 3.1 *E. coli *cells (Invitrogen). Forward and reverse attR1/attR2 recombination cassettes were identified by DNA sequencing and resulted in pBG210 and pBG211, respectively. pBG440 (pENTR-D/Topo-GFP) and pBG403 (pENTR-D/Topo-Renilla Luciferase) were constructed by PCR amplifying the eGFP gene from pIE-GFP (Clontech) with primers BG518 (5' CACCGCTAGCATGGGGATGCATGGTACCATGG) and BG519 (5' AAGTGCTAGCTTACTTGTACAGCTCGTCCATGCC) or the Renilla luciferase gene from pWN5'RucPur [18] with primers BG556 (5' CACCATGGCTAGCAAGGTGTACGACC) and BG557 (5' CTACTGCTCGTTCTTCAGCACG) and incubating the gel extracted PCR products with pENTR-D/Topo. pBG344 (pENTR-D/TOPO-mCherry) was produced by incubating the mCherry gene PCR amplified from pmCherry (Clontech) with primers BG504 (5' CACCAGATCTATGGTGAGCAAGGGCGAGGAGG) and BG505 (5' CATGAGATCTTTACCGGTGCTTGTACAGCTCGTCC) with pENTR-D/Topo. BG212 was generated by performing a LR Clonase II reaction between pBG440 and pBG210, pBG451 was generated from a LR Clonase II Reaction between pBG403 and pBG210, and pBG452 was generated from a LR Clonase II reaction between pBG344 and pBG210.

**Figure 1:**
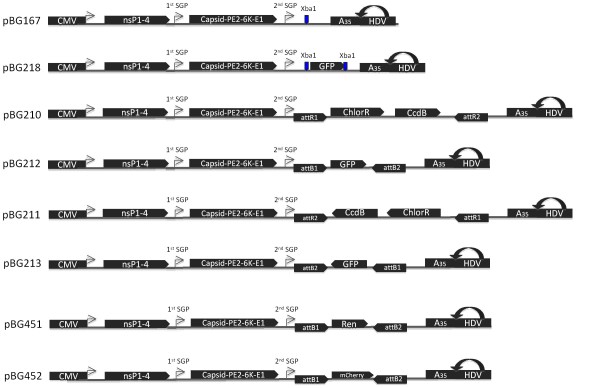
**Plasmid constructs**. The full-length double subgenomic Sindbis genome TE3'2J was engineered behind a cytomegalovirus promoter (CMV), and a 3' hepatitis delta virus ribozyme (HDV) was added after a 35 base adenine repeat (A35) to cleave the viral RNA from the over length transcript. pBG167 possess a unique XbaI site after the second SGP, and is analogous to pTE3'2J [17]. pBG218 is pBG167 with GFP inserted at XbaI site via ligation between the unique XbaI site in pBG167 and engineered NheI sites flanking the GFP ORF. pBG210 and pBG211 have the attR1/attR2 Gateway Recombination Cassettes ligated into the pBG167 XbaI site in forward and reverse orientation, respectively. pBG212 is the product of a LR Clonase reaction between pBG210 and pBG440 and places GFP in the sense orientation. pBG213 is a recombination between pBG211 and pBG440, and places GFP in the reverse orientation. pBG451 is the product of a recombination between pBG210 and pBG403, and places Renilla luciferase in the sense orientation. pBG452 is the product of a recombination between pBG210 and pBG344 and places mCherry in the sense orientation.

All viral sequences in pBG167 plasmid were verified with a panel of Sindbis-specific primers. Inserts ligated into the unique XbaI site in pBG167 were sequenced with primers BG 583 (5' CTAGATAAATGGTTAATATAGT) and BG626 (5' CACCTCTAGACCATGGATCC) to verify sequence and orientation. PCR products that were Topo-cloned into pENTR-D/Topo were sequenced with M13_-20 _and M13 Reverse primers. All Gateway attR1/attR2 containing plasmids were grown on ccdB resistant DB3.1 *E. coli *cells (Invitrogen), and all other plasmids were grown in DH5α *E. coli *cells. Diagrams of each virus expression construct are provided in Figure 1.

### Cell Culture and Transfection

Baby Hamster Kidney (BHK) and Vero cells were maintained in Hyclone DMEM supplemented with 10% fetal bovine serum (FBS), 5% Pen/Strep, and 5% L-Glutamine [18]. These mammalian cells were kept in a 37°C incubator with 5% CO_2_. BHK cells were plated in 6-well plates for transfection with Lipofectamine 2000 (Invitrogen). Cells were transfected at 60% confluency with 125 ng DNA per well following the manufacturer's recommendations. Transfection media was removed and replaced with fresh media 6-8 hours post transfection. *Aedes albopictus *C6/36 cells were cultured in L-15 medium with 10% FBS, 5% Pen/Strep, and 5% L-Glutamine and were maintained at 28°C [19].

### Plaque Assays and Growth Curves

Viral titers were determined using plaque assay titrations on BHK cells as described previously [20]. BHK cells seeded on 24 well tissue culture plates were infected with serial dilutions of virus samples for 1 hour at 37°C, and then an agarose nutrient overlay was added. Cells were maintained at 37°C for 3 days for visible plaques to develop. On day 3, Thiazolyl Blue Tetrazolium Blue (MTT) at 5 mg/ml in PBS was added to the overlay to visualize plaques and incubated at 37°C for 12 hrs. Viral plaques were counted and titers determined as plaque forming units (PFU)/ml.

P0 (transfection initiated) and P1 (virus initiated) growth curves were performed in 6-well plates. For P0 growth curves, BHK cells were transfected as described above. The cells were washed with media to remove excess transfection complexes, and 500 μl samples were collected at 4, 8, 12, 24, 36, 48, 60, and 72 hours post transfection. Sample volumes collected were replaced with fresh media to maintain a total volume of 2 ml. Aliquots of samples were stored at -80°C until titration or infection. For P1 growth curves, titered P0 derived virus (48 hr post-transfection) was added to BHK or Vero cells at MOI = 0.1 or 0.01 as indicated. Samples were collected and analyzed as described for P0 growth curves. Each growth curve was replicated three times, and average titers and standard error calculated. Data was graphed using Microsoft Excel.

### GFP, mCherry, and Renilla Luciferase expression

GFP and mCherry expressing cells were imaged on an inverted Nikon Photopt fluorescence microscope with a CoolSnap CCD camera using either 488 nM/535 nM filters for GFP detection, 560 nM/630 nM filters for mCherry detection, or phase contrast for cell imaging. Image contrast was adjusted for all images equally using ImageJ software. Luciferase assays were performed using Viviren Live Cell Renilla Luciferase Reagent (Promega) in white opaque 96-well plates. Briefly, at the indicated times media was removed from the infected wells and replaced with 25 μL DMEM supplemented with 37 μg/ul Viviren reagent. The plates were incubated at 37°C for 10 minutes, then relative light units (RLU) in each well were determined on a Victor 3 V Multimode platereader (Perkin Elmer). All experiments were performed at least 3 independent times, and averages and standard errors are reported.

## Results and Discussion

### Production of infectious Sindbis viruses from plasmid transfections

**Figure 2:**
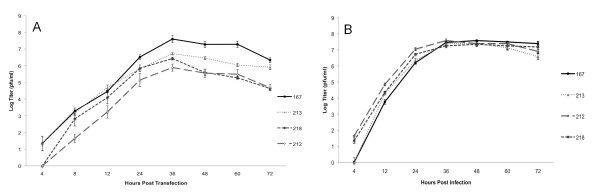
**Production of Infectious Sindbis Viruses**. A) P0 viral titers from plasmid transfected BHK cells. 125 ng of plasmid DNA transfected with Lipofectamine 2000 reagents. Media samples were collected at the indicated times and viral titers were determined by plaque assays as plaque forming units (PFU)/ml. B) P1 viral titers in BHK cells. BHK cells were infected at MOI = 0.01 with 48 hr P0 viruses, then samples were collected at the indicated times and viral titers were determined by plaque assay.

To test if infectious Sindbis virus could be produced from plasmid pBG167 (Figure 1), 125 ng of pBG167 plasmid was transfected with Lipofectamine 2000 into a single well of BHK cells in a 6-well plate. 4 hrs post transfection the cells were washed with media to remove excess transfection complexes, and media samples were collected at the indicated times post transfection (Figure 2A). Viral growth kinetics and maximal viral titers are similar to TE3'2J produced from RNA transcripts [9,21], indicating that a simple transfection of Sindbis virus expression plasmid produces similar amounts of virus as traditional production methods.

Once we determined that infectious Sindbis virus could be produced directly from transfected pBG167 plasmid, we generated a series of constructs with GFP inserted behind the 3' SGP either by ligation into the XbaI site (pBG218) or by recombination into a Gateway attR1/attR2 recombination cassette in plasmids pBG210 or pBG211 (pBG212, and pBG213). These plasmids were stably propagated in *E. coli *in the pcDNA3.1 vector backbone with average plasmid miniprep yields of 100 ng/μl (data not shown), and were able to produce infectious virus in passage 0 (P0) growth curves (Figure 2A). We observed that the maximal titers for each GOI containing clone was lower than pBG167-derived virus, which has been reported previously from transcription-derived TE3'2J viruses [9]. To determine if the plasmid derived viruses were able to replicate in a second passage (P1), we infected BHK cells with each P0 virus at MOI = 0.01 and determined growth kinetics (Figure 2B). The P1 viral growth kinetics and maximal titers were much more uniform that the P0, which may be accounted for by differing molar amounts of plasmid being transfected in the P0 samples due to differing plasmids sizes. Therefore the system was able to produce infectious virus.

### Reporter Gene Expression

**Figure 3:**
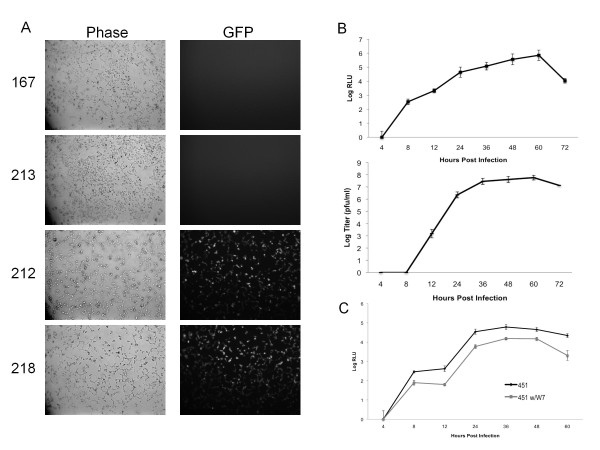
**Expression of Heterologous Genes from Recombinant Sindbis Viruses**. A) Fluorescence microscopy of BHK cells infected with GFP or non-GFP expressing viruses. BHK cells were infected with P0 virus at MOI = 0.01. At 48 hrs post infection, phase contrast and GFP fluorescence was detected at 10× magnification. B) Expression of Renilla Luciferase from pBG451. BHK cells were infected with P0 derived pBG451 virus at MOI = 0.1, and media samples were collected for plaque assay at the indicated times. In parallel, Renilla luciferase activity was detected in pBG451 infected cells using Viviren Live Cell Renilla Luciferase Reagent as described in the Materials and Methods section. C) Reduction of Renilla luciferase activity in the presence of a viral replication inhibitor. HEK 293T cells were infected in triplicate with P0 derived pBG451 virus at MOI = 0.1. At 4 hrs post infection cells were either mock treated or treated with 20 uM W-7. Renilla luciferase assays were performed at the indicated times as described in the Materials and Methods section. All reported values are the average of three independent experiments.

We next tested reporter gene expression from each virus. pBG212 and pBG218 derived virus both produced GFP expression as visualized by fluorescence microscopy, whereas pBG213 derived virus with GFP inserted in reverse orientation and pBG167 did not express GFP (Figure 3A). We also tested the kinetics of GOI expression using P0 Renilla luciferase expressing virus derived from pBG451 on BHK cells. The kinetics of Renilla luciferase expression in the P1 infection closely followed the kinetics of virus production in growth curve assays (Figure 3B), indicating that viral replication and reporter gene expression were closely linked. The slight delay in PFU production as compared to Renilla luciferase activity at 8 hrs post infection likely reflects that the subgenomic promoters are functioning and producing Renilla luciferase and viral structural proteins, but the virions had not had time to assemble into infectious viruses at that point. To verify that reporter gene expression is sensitive to a known viral replication inhibitor, we treated cells with the cellular calmodulin kinase inhibitor W-7 (N-(6-Aminohexyl)-5-chloro-1-naphthalenesulfonamide hydrochloride) that had previously been shown to interfere with viral packaging [22]. 20 μM W-7 treatment reduced reporter gene expression by about 1 log in P1 infections as compared to untreated samples (Figure 3C), demonstrating that Renilla luciferase expression was linked to viral replication.

### Replication of plasmid derived virus in various cell lines

**Figure 4:**
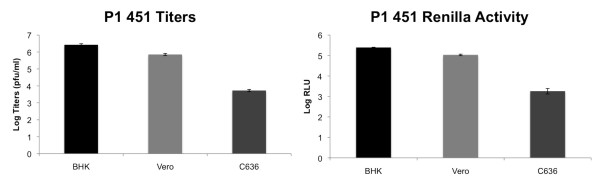
**Replication and Heterologous Gene Expression in Cell Lines from Different Species**. BHK (murine), Vero (primate) and C636 (mosquito) cell lines were infected with P0 derived pBG451 virus at MOI = 0.1. Renilla luciferase assays and plaque titrations were performed in triplicate on each cell line at 48 hours post infection.

Sindbis is known for broad tropism and is able to infect different species with relatively similar efficiencies. We determined if plasmid-derived virus was able to replicate well in cell lines other than BHK cells. Renilla luciferase expressing pBG451 virus (P0) was used to infect BHK (murine), Vero (primate), and C6/36 (mosquito) cell lines at MOI = 0.1, and viral titers and Renilla luciferase activity were assessed at 48 hrs post infection (P1) (Figure 4). BHK cells and Vero cells produced similar titers 48 hrs post infection (~1 × 10^6 ^PFU/ml), and showed similar Renilla luciferase signals at 48 hrs (~1 × 10^5 ^RLU). C6/36 cells produced lower titers and Renilla luciferase signal than BHK and Vero cells at 48 hrs post infection, though this difference may be due to slower growth kinetics in invertebrate cell lines. These data indicate that the P0 virus was able to effectively infect and replicate in both murine, primate, and mosquito cell lines, and that the Renilla luciferase reporter gene was expressed in each cell line during the P1 infection.

### Stability of Reporter Gene Expression

**Figure 5:**
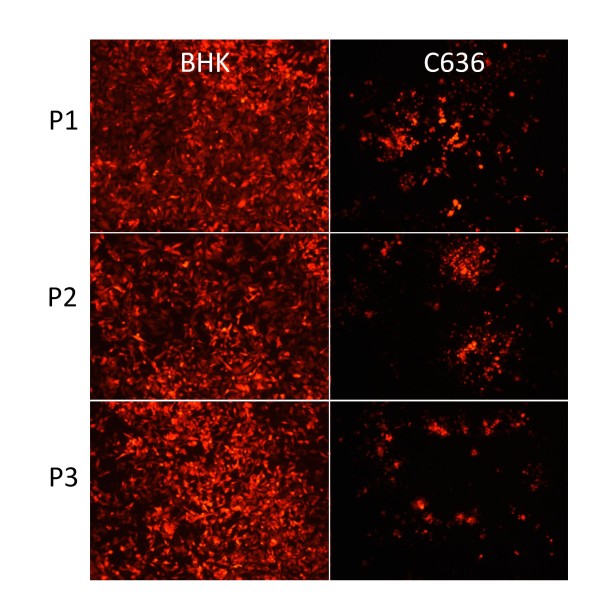
**Stability of Reporter Gene Expression**. BHK or C636 cell lines were infected with P0 derived pBG452 virus at MOI = 0.01 for 48 hrs, then media was collected and titered. This process was repeated for a total of 3 passages. After the 3^rd ^passage, BHK or C6/36-derived virus from each passage was added to BHK or C6/36 cells at MOI = 0.01 and incubated for 28 (BHK) or 86 (C6/36) hrs. mCherry fluorescence was detected at the indicated times by fluorescence microscopy.

To test the stability of the att-containing viruses, we passaged the mCherry containing pBG452 virus 3 times in either BHK or C6/36 cells using MOI = 0.01 for each infection. We collected media after 48 hrs for each passage, and after the 3^rd ^passage we infected naïve BHK or C6/36 cells with virus from passage at MOI = 0.01. We observed that each cell type showed equal fluorescence with each passage at 28 hrs post-infection (BHK) and 86 hrs post-infection (C6/36) (Figure 5), indicating that the att-flanked GOI remained stable over the course of at least 3 passages in both BHK and C6/36 cells.

The typical sequence of events for production of a GOI expressing TE'32J virus takes approximately 3-4 weeks. Cloning and inserting the GOI 3' of the viral subgenomic promoter usually takes ~2 weeks from the PCR amplification step and requires screening clones for insert orientation. Once a clone has been identified and the GOI sequenced, the production of infectious RNA takes 2-3 additional days due to preparation of linearized maxiprepped plasmid DNA for *in vitro *transcription and electroporation of RNA transcripts into ~2-10 × 10^6 ^cells using expensive electroporation systems. Additionally, because RNA is being used, extra precautions to reduce RNase degradation of transcripts must be used. Therefore, the traditional production of GOI expressing TE3'2J can be time consuming and somewhat expensive.

Our system circumvents many of the problems associated with production of GOI expressing TE3'2J viruses. GOI PCR products can be rapidly cloned into attL containing pENTR-D/Topo vectors, then the GOI can be recombined into pBG210 (forward) or pBG211 (reverse) TE3'2J expression plasmids with high efficiency. In our hands this process take about 1 week and is highly efficient. The pENTR-D/Topo vector and LR Clonase II systems from Invitrogen are relatively expensive, but each reaction can be scaled down to reduce costs and extend the number of reactions that can be performed. We have successfully recombined short hammerhead ribozymes (67 bp) and the large Firefly luciferase gene (1.6 Kb) into pBG210 (data not shown), indicating that a wide range of insert sizes can be accommodated. Once the GOI containing virus expression plasmid has been constructed, virus is produced by transfecting a small amount of the plasmid into BHK cells using common transfection agents and 2 × 10^5 ^cells in a single well. Virus is produced within a few days with minimal effort. The format for transfection can be adjusted from 96-well plates to T_150 _flasks as needed, making virus production very flexible and rapid. GOI expression was verified from several viruses, including viruses that produce GFP and Renilla luciferase (Figures 3 and 4). The kinetics of Renilla luciferase expression closely mirrored viral replication kinetics, indicating that the GOI is stable and can be used as a readout for viral replication as previously described [7-9].

## Conclusions

In this report we describe the construction and characterization of a new Sindbis (TE3'2J) virus production system. The virus expression plasmids we describe have several features that make them useful for rapidly generating Sindbis viruses that express genes of interest. We have developed a system by which fully infectious reporter gene expressing virus can be produced simply by transfecting a small amount of virus expression plasmid into cultured mammalian cells. Genes of interest can be rapidly incorporated into viruses in specific orientations via Gateway recombination. High-titer virus produced from our system can infect multiple cell types in culture and maintain reporter gene expression. The ease of cloning and specificity of insert orientation would make this system ideal for generating libraries of infectious viruses expressing randomized trans-cleaving ribozymes or inverted cDNA libraries to screen for host genes that are involved in viral replication or antiviral responses. In addition, the ability to launch virus production directly in cells with stable plasmid DNA may open up the possibility of using these constructs for stable Sindbis virus vaccines that can be launched via plasmid injection that may provide more robust immune responses than non-spreading replicon vaccines.

## Competing interests

The authors declare that they have no competing interests.

## Authors' contributions

JS constructed and tested the virus expression plasmids, helped design the study, and helped write the manuscript. BRH and SBCL helped perform plaque and luciferase assays during. KEO and BJG provided funding for the manuscript, and BJG helped write the manuscript and design the overall project. All authors have read and approved this manuscript.
